# Importin 7 and Nup358 Promote Nuclear Import of the Protein Component of Human Telomerase

**DOI:** 10.1371/journal.pone.0088887

**Published:** 2014-02-20

**Authors:** Cornelia Frohnert, Saskia Hutten, Sarah Wälde, Annegret Nath, Ralph H. Kehlenbach

**Affiliations:** Institute of Molecular Biology, Faculty of Medicine, University of Göttingen, Göttingen, Germany; University of Nebraska Medical Center, United States of America

## Abstract

In actively dividing eukaryotic cells, chromosome ends (telomeres) are subject to progressive shortening, unless they are maintained by the action of telomerase, a dedicated enzyme that adds DNA sequence repeats to chromosomal 3′end. For its enzymatic function on telomeres, telomerase requires nuclear import of its protein component (hTERT in human cells) and assembly with the RNA component, TERC. We now confirm a major nuclear localization signal (NLS) in the N-terminal region of hTERT and describe a novel one in the C-terminal part. Using an siRNA approach to deplete several import receptors, we identify importin 7 as a soluble nuclear transport factor that is required for efficient import. At the level of the nuclear pore complex (NPC), Nup358, a nucleoporin that forms the cytoplasmic filaments of the NPC, plays an important role in nuclear import of hTERT. A structure-function analysis of Nup358 revealed that the zinc finger region of the nucleoporin is of particular importance for transport of hTERT. Together, our study sheds light on the nuclear import pathway of hTERT.

## Introduction

The molecular mechanisms of DNA-polymerases result in incomplete replication of DNA at telomeres, the ends of linear chromosomes. As a consequence, DNA-ends were predicted to shorten with each round of replication, leading to a limited number of possible somatic cell divisions [1]. For continued proliferation of cells, a mechanism is required that counteracts the progressive loss of DNA-sequences. The major mechanism involves the enzyme telomerase, a ribonucleoprotein with reverse transcriptase activity that adds telomeric DNA-repeats to the 3′ends of chromosomes (for review see [2], [3]). In this reaction, the RNA-component of telomerase (TERC) functions as a template that is reverse transcribed into the characteristic telomere repeat sequence (TTAGGG in vertebrates), elongating the DNA-3′-end. The protein component of telomerase (hTERT in humans; 1132 amino acids) contains characteristic catalytic motifs also known from retroviral reverse transcriptases [4]. The TERT-gene is expressed in germ cells and embryonic tissues but is largely turned off in most adult tissues [2]. Many cancer cells, by contrast, reactivate its expression, allowing indefinite growth without concomitant loss of chromosome ends. The TERC-gene, on the other hand, is rather constitutively expressed but can be upregulated in cancer cells as well. In order to fulfill its enzymatic function, telomerase must be imported into the nucleus and a number of studies suggested that regulation of nucleocytoplasmic shuttling of the protein is a means to control its activity [5]–[7]. In the nucleus, several steps lead to the assembly of active telomerase ribonucleoproteins (for review see [8]).

Nucleocytoplasmic transport in general occurs through the nuclear pore complexes (NPCs) that are embedded between the inner and the outer nuclear membrane. NPCs are composed of a set of ∼30 different nucleoporins (Nups), which occur in a copy number of eight or multiples of eight [9], [10]. Soluble transport receptors of the importin β-superfamily interact with Nups and mediate the translocation of import or export complexes across the NPC. In many cases, the receptors bind to characteristic phenylalanine-glycine- (FG-) motifs, found on a subset of nucleoporins, the FG-Nups (for review see [11], [12]). Transport receptors recognize transported cargo molecules containing characteristic nuclear localization- or nuclear export signals (NLSs and NESs, respectively). The best-described nuclear import receptor is the dimer of importin α and importin 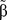 Importin α serves as an adapter protein that binds the classic NLS (cNLS), which is characterized by either one (monopartite NLS) or two (bipartite NLS) stretches of amino acids that are enriched in basic residues [13]. Importin β interacts with a region at the N-terminus of importin α and the trimeric, cargo-containing complex is then imported into the nucleus. Here, the small GTP-binding protein Ran in its GTP-bound form interacts with importin β, leading to dissociation of the import complex. RCC1, the nucleotide exchange factor for Ran, is largely bound to chromatin, resulting in a high concentration of nuclear RanGTP (for review see [11], [12]). Similar mechanisms govern import by other members of the importin β-superfamily with other, often ill-defined nuclear localization signals. Examples are transportin, recognizing a characteristic PY-motif in imported proteins [14] and importin 5, importin 7 and importin 9, which, among others, transport histones and ribosomal proteins into the nucleus [15]–[17]. All importin β-like transport receptors seem to leave the nucleus in a complex with RanGTP. In the cytoplasm, the GTPase-activating protein RanGAP then promotes GTP-hydrolysis on Ran [18], leading to the dissociation of this complex. Interestingly, a large proportion of RanGAP associates with the giant nucleoporin Nup358/RanBP2, the major component of the cytoplasmic filaments of the NPC [19], [20].

With respect to telomerase, surprisingly little is known about its nuclear import pathway. Very recently, a bipartite NLS, as it is characteristic for the importin α/β-pathway, was described in hTERT [21]. The relevant transport receptor, however, was not explicitly identified in this study. In yeast, the *S. cerevisiae* protein Mtr10, which is related to a transportin variant, was identified as an important transport factor for the RNA component of yeast telomerase [22]. For nuclear export of telomerase, on the other hand, the major export receptor CRM1 was suggested to play a role under certain conditions [6], [23].

In this study, we confirm the activity of the previously identified NLS of hTERT and describe a second, albeit less active, NLS in the C-terminal part of the protein. Furthermore, we identify the nuclear import receptor importin 7 as a major factor that mediates transport of hTERT into the nucleus. Efficient transport also requires the cytoplasmic nucleoporin Nup358, which was previously implicated in nuclear import of a subset of proteins [24].

## Materials and Methods

### Cell Culture and Transfections

HeLa-P4 cells [25] cells and HEK 293T cells were grown in DMEM (GIBCO) containing 4500 mg/l glucose, 10% fetal calf serum, 2 mM glutamine. Cells were transiently transfected using calcium phosphate [26] or Polyfect (Qiagen).

### RNA Interference

Cells were transfected with small interfering RNAs (siRNAs) from Ambion, Eurofins or Santa Cruz (importin 7, NM_006391, GAUGGAGCCCUGCAUAUGA; importin 7, alternative, UGAUGACCUUACCAAUGUA; importin 9, UCACUGAGGAGCAGAUUAA, NM_018085; importin β, ACAGUGCCAAGGATTGTTA, NM_002265; Nup358, CACAGACAAAGCCGUUGAAUU, NM_006267; transportin, pool of three specific siRNAs, Santa Cruz, Sc-35737, NM_002270.3; non-targeting, GAGCUUCAACUAACAGGAATT) at a final concentration of 100 nM, using Oligofectamine (Invitrogen), according to the instructions of the manufacturer. 72 h after siRNA treatment, cells were plated on coverslips, transfected with reporter protein constructs of interest and analyzed after incubation for additional 20–24 h. For depletion of Nup358, cells were transfected twice with the siRNA, as described previously [24], [27].

### Plasmids

The plasmid coding for myc-EZI was kindly provided by Tohru Itoh [28]. Plasmids coding for Nup358-fragments [24] and reporter proteins NES-GFP_2_-M9 [29] and NES-GFP_2_-cNLS [30] were described previously. hTERT-expression constructs were derived from an hTERT-myc plasmid, kindly provided by Judith Haendeler [6]. Fragments of TERT as well as the full-length sequence were cloned into pEGFP-N1 (full-length, HindIII-EcoRI) or pEGFP-C2 (fragments, HindIII-BamHI) vectors using appropriate PCR-primers. To obtain an siRNA-insensitive expression plasmid for importin 7, a construct coding for HA-tagged importin 7 from *Xenopus laevis* [24] was used as starting material. Point mutants were generated by site directed mutagenesis, using oligonucleotides 5′-AAAGACGGCGCTCTACAGATGA and 5′-TCATCTGTAGAGCGCCGTCTTT. 5′-AGTCTGCCGTTGCCCGCGGCGCCCAGGCGT and 5′-ACGCCTGGGCGCCGCGGGCAACGGCAGACT were used for the hTERT K236AR237A-mutation and 5′-TTCCGCAGAGAAGCGGCGGCCGAGCGT and 5′-ACGCTCGGCCGCCGCTTCTCTGCGGAA for the hTERT K649AR650A-mutation. All constructs were verified by sequencing (GATC, Konstanz).

### Protein Expression

GST-Nup358-fragment [24], importin 7 [15] and RanQ69L [31] were expressed and purified as described before. GTP-loading of Ran was as described before [32].

### Binding Assays

5 µg of GST-tagged proteins were immobilized on 10 µl of glutathione-sepharose beads. After blocking of non-specific binding sites with 1% BSA, beads were incubated with 5 µg His-importin 7 in a final volume of 1 ml of binding buffer (50 mM Tris-HCl, pH 7.3, 200 mM NaCl, 1 mM MgCl_2_, 5% glycerol, 1% BSA, 1 mM DTT, 1 µg/ml each of aprotinin, leupeptin and pepstatin) for one h at 4°C. Some reactions also contained 2.5–10 µg RanQ69L, loaded with GTP. After the reaction, beads were washed three times with binding buffer without BSA. Bound proteins were analyzed by SDS-PAGE, followed by Coomassie-staining.

### Antibodies

The antibody against importin 7 was raised in rabbits against a peptide from the C-terminal region of the protein (CLADQRRAAHESKMIEKHG) and affinity-purified. The antibody against importin β was raised in rabbits against the full-length protein and affinity purified. Antibodies against Nup358 were described previously [30]. Rabbit-anti-tubulin was from Proteintech (11224-1-AP). For the detection of tagged proteins, mouse-anti-myc (clone 9E10, Serotec), mouse-anti-HA (clone 16B12, Covance) and rabbit-anti-GFP (Santa Cruz) were used. For immunofluorescence, secondary antibodies from donkey, coupled to Alexa 488, Alexa 594 or Alexa 647 were used (1∶2000; Molecular Probes). For immunoblotting, HRP-coupled donkey anti-rabbit IgG (Dianova) was used as secondary antibody.

### Immunoprecipitation

HEK 293T cells were transfected with plasmids coding for GFP or TERT-GFP. After 48 h, cells were trypsinized, washed with PBS and lysed in GFP-buffer (10 mM Tris-HCl, pH 7.2, 150 mM NaCl, 0.5 mM EDTA, 0.5% NP-40, 1% BSA, 2 mM DTT, 1 mM PMSF, 1 µg/ml each of aprotinin, leupeptin and pepstatin; 200 µl/10-cm plate). The lysate was cleared by centrifugation at 16,000 g for 10 min and diluted with GFP-buffer lacking NP-40 to yield a final concentration of the detergent of 0.2%. 8 µl of pre-equilibrated GFP-trap beads (Chromotek) were added. After 2.5 h at 4°C, beads were washed three times with GFP-buffer lacking NP-40 and BSA. Bound proteins were analyzed by SDS-PAGE, followed by immunoblotting.

### Immunofluorescence

Immunofluorescence staining was essentially performed as described [27]. Cells were analyzed by fluorescence microscopy using a Zeiss Axioskop2 microscope and AxioVision software or a confocal microscope (510-Meta, Carl Zeiss, Jena, Germany) equipped with an argon laser and a 63x Plan-Neofluar 1.3NA water-corrected objective. Pictures were processed using Adobe Photoshop.

For quantification of subcellular localization of reporter proteins, cells were grouped into three categories: N>C (majority of the reporter protein in the nucleus); N = C (equal distribution between nucleus and cytoplasm); C>N (predominant cytoplasmic localization). Quantification was performed from at least three independent experiments, counting ∼100 cells with similar expression levels. Statistical significance of the data was analyzed by a two-tailed, heteroscedastic students t-test. p-values <0.05 were considered as biologically significant.

### Live Cell Imaging

24 h after transfection of hTERT-GFP constructs, HeLa cells grown on LabTec-chambers (Nunc) were transferred to CO_2_-independent medium (Gibco). FLIP-experiments were carried out at 37°C in a temperature controlled chamber attached to a confocal microscope (510-Meta, Carl Zeiss, Jena, Germany) equipped with an argon laser and a 63x Plan-Neofluar 1.3NA water-corrected objective. For analysis of nuclear import during 146 seconds, a nuclear region was subjected to 180 bleach intervals with the argon laser set to 100%. After each interval, the fluorescence emission was collected during a period of 154 ms at 2% laser intensity with the pinhole set to 2.0 airy units. Signal intensities were analyzed from cytoplasmic regions of a bleached (F_bc_) and a neighboring unbleached cell as a reference (F_rc_), and a background region (F_bg_). Cytoplasmic fluorescence intensities for each time point were expressed as F(t) = (F_bc_–F_bg_)/(F_rc_–F_bg_), normalizing the pre-bleach fluorescence to 1. 10–20 cells were analyzed per condition.

## Results

### Several Basic Regions Contribute to Nuclear Import of hTERT

**Figure 1:**
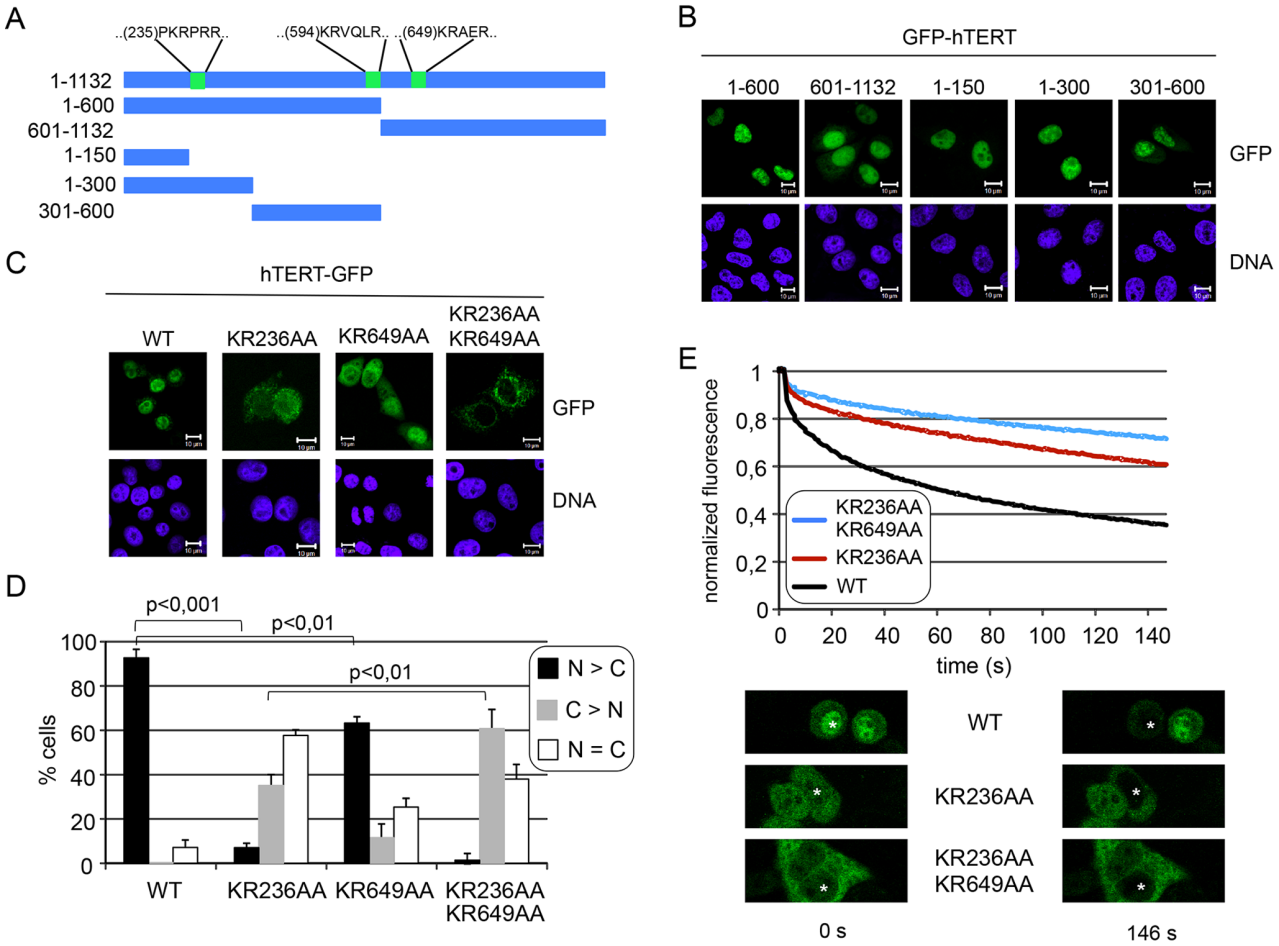
Analysis of potential NLSs in hTERT. A, schematic representation of GFP-tagged full-length hTERT and hTERT fragments used in this study. Putative NLSs are indicated. B, hTERT-fragments accumulate in the nucleus. HeLa cells were transfected with hTERT constructs as indicated, fixed and analyzed by confocal microscopy. C, HeLa cells were transfected with constructs coding for full-length hTERT-GFP or putative NLS-mutants, as indicated and analyzed by confocal microscopy. B, C, bars, 10 µm. D, Quantification of the results in C. Error bars show the standard deviation from the mean of three independent experiments. E, Nuclear import of wild type, GFP-tagged hTERT and two NLS-mutants was analyzed by FLIP in living cells. Cells with similar expression levels were chosen for the analysis. Examples for individual cells before and after the analysis are shown at the bottom. Asterisks depict the bleached nuclear region. The graphs (top) show the mean loss in fluorescence in three independent experiments, analyzing a total of 45 cells per condition. Error bars were omitted for clarity (see Fig. S1 for an example of a FLIP-experiment with error bars).

As a first step towards the elucidation of the nuclear import pathway of hTERT, we analyzed the subcellular localization of tagged full-length protein and of fragments thereof (Fig. 1A–C). Different versions of full-length hTERT-HA, myc-hTERT (not shown) or hTERT-GFP (Fig. 1C) were detected almost exclusively in the nucleus. Likewise, all tested hTERT-fragments, fused to GFP, were found in the nuclear compartment (Fig. 1B). The small N-terminal fragments (1–150, 1–300, 301–600) were also tested as fusion proteins linked to GFP-GFP to increase the molecular weight of the proteins and to hamper passive diffusion across the NPC. These larger proteins also accumulated in nuclei of transfected cells, similar to their smaller counterparts (data not shown). These results suggested that it is not a single, isolated NLS that allows import of hTERT into the nucleus. Furthermore, hTERT-fragments might interact non-specifically with cellular proteins that are actively imported into the nucleus. We therefore decided to analyze the hTERT sequence with respect to putative NLSs. hTERT is a rather basic protein with a total of 165 lysine and arginine residues and an isoelectric point of 10.5. Using the Eukaryotic Linear Motif Resource for Functional Sites in Proteins (ELM; http://elm.eu.org), one region was identified that is enriched in basic amino acids, corresponding either to a bipartite or a monopartite NLS (amino acids 222–240 of the full-length sequence). Of particular interest was the short peptide PKRPRR at position 235–240, listed by the program as a “variant of the classical basically charged NLS” (Fig. 1A). In light of the difficulties with protein fragments, we performed site-directed mutagenesis on full-length hTERT-GFP, exchanging amino acids 236 and 237 to alanines (KRPRR to AAPRR). Upon transfection into HeLa cells, a clear difference in the subcellular localization of the mutant compared to the wild-type protein was observed (Fig. 1C, D). Whereas the latter accumulated in the nucleus in 90% of transfected cells, the mutant protein accumulated in the cytoplasm or was equally distributed between the two compartments. These results suggested that the basic amino acids at position 236–237 are major determinants of the nuclear localization of hTERT. During the course of our studies, the same region in the hTERT sequence was reported to function as an NLS [21]. In our study, however, we did not observe a complete exclusion from the nucleus of the full-length protein, when characteristic basic residues were mutated to alanines. A possible explanation for this observation is the presence of additional NLS(s) in the hTERT sequence. We therefore searched the hTERT sequence for additional basic regions that might function as an NLS and exchanged the second (594–595) and the third (649–650) KR-motif to AA (Fig. 1A). The mutant full-length protein hTERT-GFP KR594AA was found in the nucleus of over-expressing HeLa cells, very similar to the wild-type protein (data not shown). The other mutant, hTERT-GFP KR649AA, however, showed a significant shift towards the cytoplasm (Fig. 1C, D). This change was by far not as pronounced as the one we had previously observed for the first KR-mutant, hTERT-GFP KR236AA. We therefore expressed a mutant, where both KR-motifs, (236–237 and 649–650) were changed to alanines. In this quadruple mutant, the majority of cells excluded full-length hTERT-GFP from the nucleus, enhancing the effect of the original KR236AA-mutation (Fig. 1C, D).

The results described so far were obtained using fixed cells. To corroborate our findings and to further analyze the dynamics of nuclear import, we performed fluorescence loss in photobleaching (FLIP) experiments in living cells. GFP-tagged wild type and mutant hTERT were expressed in HeLa cells and the fluorescence in the nuclei of expressing cells was repeatedly bleached. The rate of loss in fluorescence intensity in the cytoplasm of bleached cells was taken as a measure for the efficiency of nuclear import of the fusion proteins. As shown in Fig. 1E, the mutation at position 236–237 (KR236AA; red curve) clearly reduced the rate of nuclear import of hTERT-GFP. This effect was slightly enhanced in the quadruple mutant where also K649 an R650 were mutated to alanines (blue curve), in line with our observations in Figs. 1C and D. Together, our results suggest that the KR-motif at position 236–237 is a major determinant of the nuclear localization of hTERT. Additional sequences (e.g. the KR-motif at position 649–650) seem to contribute to efficient import of hTERT into the nucleus.

### Importin 7 is a Major Nuclear Import Receptor for hTERT

**Figure 2:**
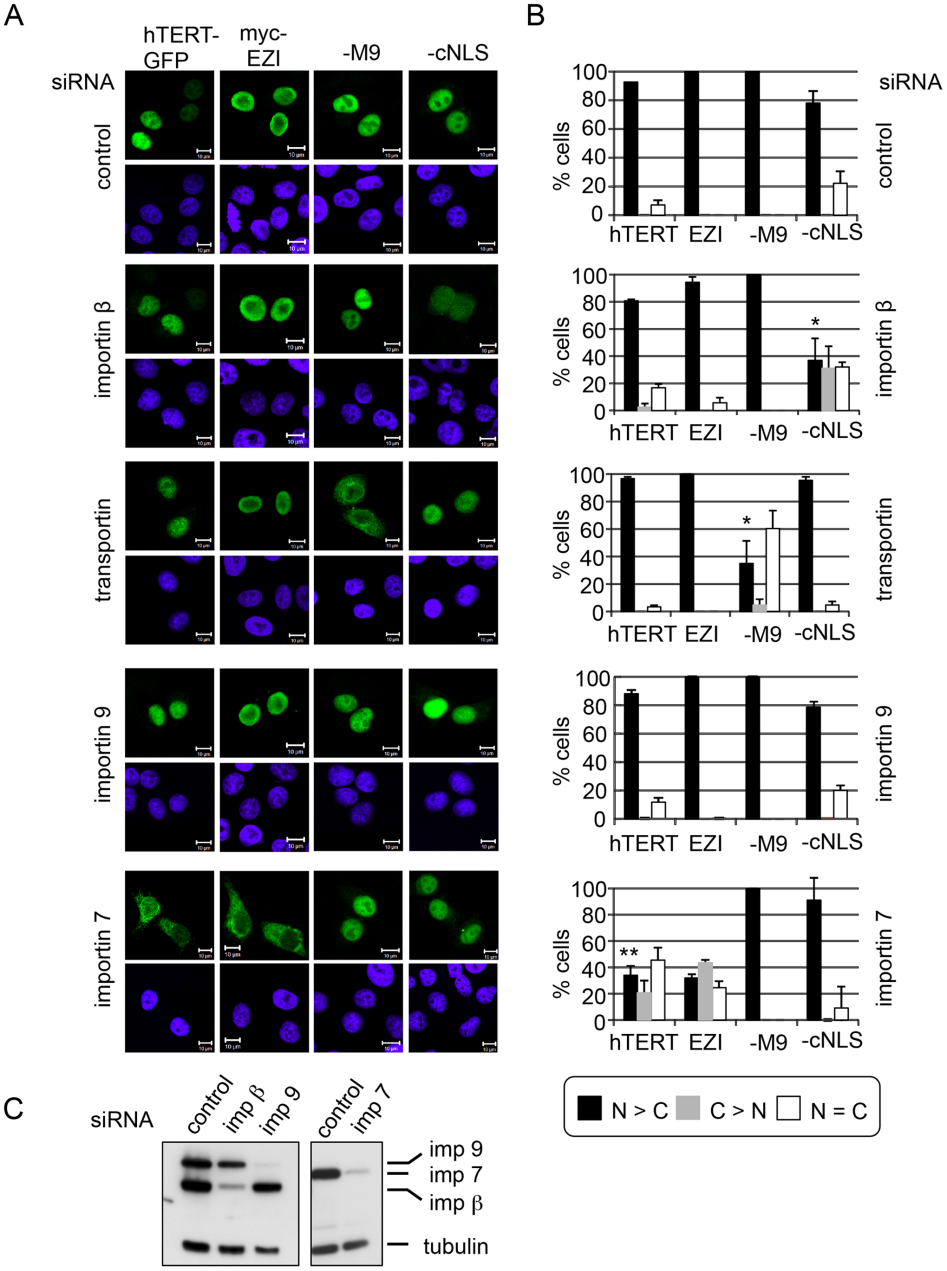
siRNA-approach to identify the import receptor for hTERT. A, HeLa cells were treated with a control siRNA or with siRNAs against importin β, transportin, importin 9 or importin 7 and transfected with plasmids coding for hTERT-GFP, myc-EZI, NES-GFP_2_-M9 or NES-GFP_2_-NLS, as indicated. Cells were fixed and analyzed by fluorescent microscopy. EZI was detected using an antibody against the myc-tag. Bars, 10 µm. B, Quantification of the results shown in A. Error bars depict the standard deviation from the mean of at least three independent experiments. Asterisks indicate p-values <0,02 (*) or <0,002 (**), compared to the control. C, Lysates of control cells and importin β-, importin 9- or importin 7-depleted cells were analyzed by immunoblotting. Tubulin was used as a loading control.

The search algorithm that is used by ELM identifies classical NLSs, i.e. sequences that are recognized by the import receptors importin α/β. Other members of the importin β- superfamily, however, interact with similar, often less well-described sequences that tend to be enriched in basic amino acids as well. We first tested whether depletion of importin β by specific siRNAs resulted in reduced nuclear import of hTERT. The level of depletion of importin β was determined by immunoblotting (Fig. 2C). As a positive control for reduced nuclear import, we used NES-GFP_2_-cNLS, a reporter protein whose nuclear import depends on the importin α/β-dimer. It is mostly nuclear under steady-state conditions, although the nuclear export signal (NES) in the reporter leads to its constant shuttling between the nucleus and the cytoplasm. As a result, inhibition of nuclear import can be readily detected. Indeed, import of NES-GFP_2_-cNLS into the nucleus was clearly inhibited in importin β-depleted cells (Figs. 2A, B). For the analysis of nuclear import of hTERT, we again used GFP-tagged versions, since several commercial antibodies against endogenous hTERT gave very inconsistent results in indirect immunofluorescence experiments in several cell lines. In contrast to the importin α/β -dependent reporter protein, transport of hTERT-GFP was hardly affected in cells with reduced importin β-levels (Fig. 2A, B), suggesting that other import receptors are involved in nuclear localization of hTERT.

Next, we used specific siRNAs to reduce the cellular levels of transportin, importin 7 and importin 9. The efficiency of the siRNA-mediated depletion of the importins was controlled by immunoblotting (Fig. 2C; importin 7 and importin 9) and/or by expression of an import cargo with a defined import receptor (transportin and importin 7). The depletion of importin 9 did not affect the nuclear localization of any of the tested import cargos (Figs. 2A, B). Depletion of transportin resulted in a clear shift of the transportin-dependent shuttling protein NES-GFP_2_-M9 from the nucleus towards the cytoplasm, whereas other reporters were not affected. Depletion of importin 7 strongly inhibited nuclear accumulation of our positive control, the transcription factor EZI, as described previously [28]. Strikingly, also the nuclear localization of hTERT-GFP was strongly reduced in importin 7-depleted cells. NES-GFP_2_-NLS and NES-GFP_2_-M9 were not affected in these cells, demonstrating the specificity of the effect.

To confirm the involvement of importin 7 in nuclear import of hTERT-GFP, we performed four types of experiments. First, we used an alternative siRNA to deplete the import receptor, directed against a different region on the mRNA. This alternative siRNA also reduced the levels of importin 7, as detected by immunoblotting. Likewise, it clearly reduced nuclear accumulation of hTERT-GFP (data not shown).

**Figure 3:**
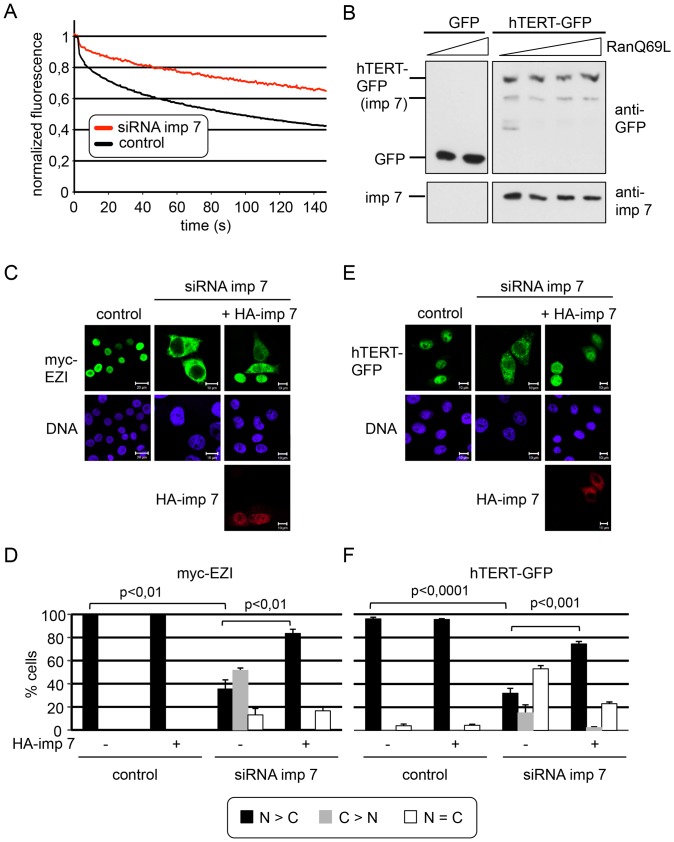
Importin 7 functions as an import receptor for hTERT. A, HeLa cells expressing hTERT-GFP that had been treated with either control siRNAs or with siRNAs against importin 7 were analyzed for the dynamics of nuclear import of the reporter protein by FLIP. The graphs show the mean loss in fluorescence in three independent experiments, analyzing a total of 45 cells per condition. Error bars were omitted for clarity (see Fig. S1 for the identical experiment with error bars). B, HEK 293 cells were transfected with constructs coding for GFP or hTERT-GFP. Cell lysates were subjected to immunoprecipitation using immobilized anti-GFP-antibodies (“GFP-trap”). Reactions contained increasing amounts (0, 10 µg/ml for GFP and 0, 2.5, 5, 10 µg/ml for hTERT-GFP) of RanQ69L that had been preloaded with GTP. Precipitated proteins (GFP, hTERT-GFP and importin 7) were analyzed by immunoblotting. The weak signal for importin 7 (imp 7) in the upper panel results from the initial detection of the import receptor on the same blot (lower panel). C-F, Overexpression of exogenous importin 7 rescues nuclear import of hTERT-GFP. HeLa cells were treated with control siRNAs or with siRNAs against importin 7 and transfected with plasmids coding for either myc-EZI (C, D) or hTERT-GFP (E, F), alone or together with a plasmid coding for HA-importin 7, as indicated. Cells were fixed and analyzed by fluorescence microscopy. myc-EZI and HA-importin 7 were detected using specific antibodies against the myc- and HA-tag, respectively. Bars, 10 µm or 20 µm (control in C). D, F, Quantification of the results shown in C and E. Error bars depict the standard deviation from the mean of at least three independent experiments.

Second, we performed live cell imaging of nuclear import by FLIP in importin 7-depleted cells, similar to the experiment described in Fig. 1E. Clearly, the depletion of importin 7 resulted in reduced import rates of hTERT-GFP in importin 7-depleted cells compared to control cells (Fig. 3A).

Third, we used a biochemical approach and immunoprecipitated overexpressed hTERT-GFP and, as a control, GFP, using the GFP-trap [33]. As shown in Fig. 3B, endogenous importin 7 (but not importin β; data not shown) co-precipitated with hTERT-GFP, but not with GFP. We also used the immunoprecipitation approach to analyze the effect of RanGTP on hTERT-importin 7 complexes. Most import complexes are efficiently dissociated by RanGTP. In the presence of GTP-loaded RanQ69L, a Ran-mutant that is defective in GTP-hydrolysis [34], however, similar amounts of importin 7 co-precipitated with hTERT-GFP, suggesting that additional mechanisms are required for disassembly of the import complex.

Fourth, (and most importantly), we designed an siRNA-insensitive expression construct for HA-tagged importin 7. The best control to exclude off-target effects in siRNA experiments is to rescue the phenotype by expression of the gene of interest from such siRNA-insensitive plasmids. For our experiments, we used importin 7 from *Xenopus laevis*, which shows 93% of sequence identity compared to the human protein, but has a base-substitution at the position that is targeted by our siRNA. Two additional substitutions were introduced by site-directed mutagenesis, rendering the resulting mRNA insensitive towards the siRNA. As shown in Fig. 3 C–F, expression of HA-importin 7 rescued nuclear import of EZI and of full-length hTERT-GFP in cells where the endogenous import receptor had been depleted. As a control, we also expressed HA-importin β in importin 7-depleted cells, but did not observe a stimulation of nuclear import of hTERT-GFP (data not shown). We conclude from these results that importin 7 is a major nuclear import receptor for hTERT. Of course we cannot exclude the possibility that other, as yet untested importins play additional roles in nuclear transport of hTERT.

### Nup358 Promotes Nuclear Import of hTERT

**Figure 4:**
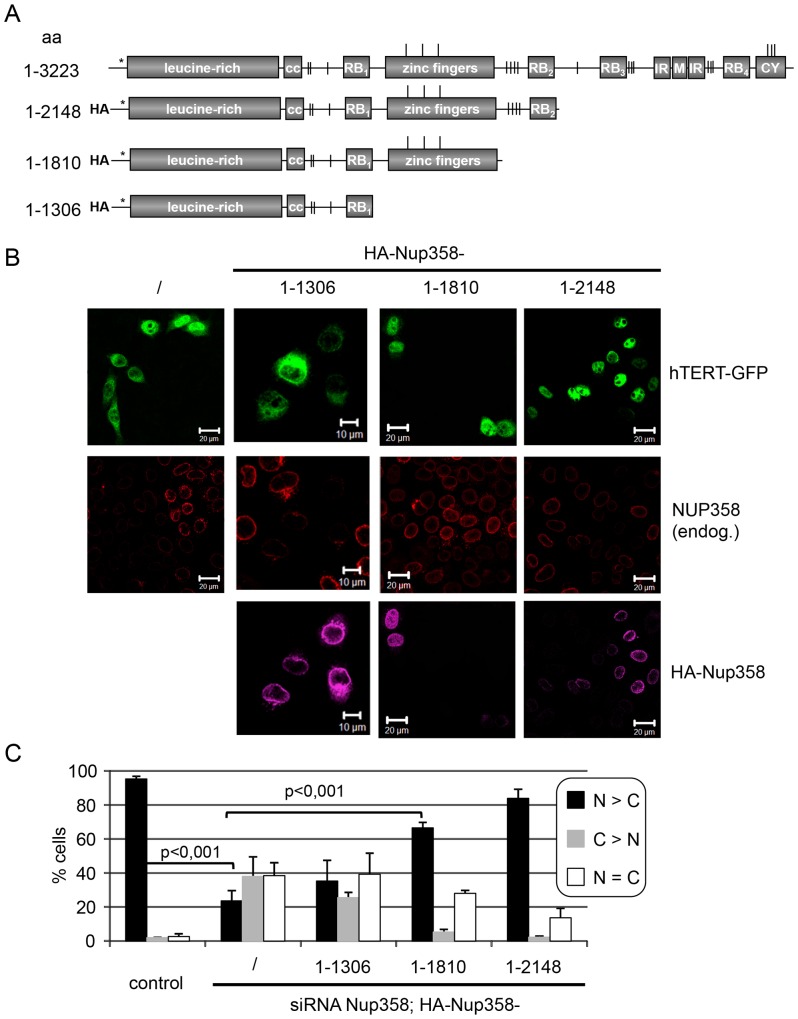
The zinc finger region of Nup358 is involved in nuclear import of hTERT-GFP. A, Schematic representation of full-length Nup358 (amino acids (aa) 1–3223) and various Nup358-fragments used as rescue constructs. cc, predicted coiled-coil region, RB1-RB4, Ran-binding domains 1–4, IR, internal repeat, M, ‘middle domain’, CY, cyclophilin domain. vertical bars, FG-motifs; asterisk, region in corresponding RNA that confers resistance to siRNA. B, HeLa cells were treated with control siRNAs (not shown; compare upper left picture for localization of hTERT-GFP in cells with high levels of endogenous Nup358) or with siRNAs to deplete Nup358, and transfected with constructs coding for hTERT-GFP and HA-tagged fragments of Nup358, as indicated. Cells were fixed and analyzed by fluorescence microscopy. Antibodies against the C-terminal region of Nup358 were used to detect the endogenous protein, whereas the anti-HA antibody was used to detect Nup358-fragments. Bars, 10 or 20 µm. C, Quantification of the results shown in B. Error bars depict the standard deviation of the mean from three independent experiments.

Nucleoporins play very general roles in nuclear import and export of proteins, as they form the permeability barrier of the NPC. Besides this common function, certain nucleoporins have been shown to affect the localization of individual proteins in a transport receptor and/or cargo-specific manner [27], [35]–[37]. We have previously shown that the giant FG-nucleoporin Nup358 (see Fig. 4A for a schematic representation) promotes the rate of nuclear import of importin α/β - and transportin cargos [29], [30]. Furthermore, a subset of nuclear proteins (∼5–10% of 200 tested proteins) require Nup358 for efficient nuclear accumulation [24]. Having established importin 7 as a soluble protein that promotes nuclear import of hTERT, we therefore set out to analyze the function of cytoplasmic nucleoporins. Regarding this rather low percentage of proteins whose nuclear localization strictly depends on Nup358, it came as a surprise that depletion of this nucleoporin resulted in a clear reduction of nuclear hTERT-GFP (Figs. 4B, C). Depletion of Nup214, another nucleoporin that localizes to the cytoplasmic side of the NPC, did not affect the localization of hTERT (data not shown). Furthermore, nuclear accumulation of EZI, our importin 7-dependent control protein, was not reduced by the depletion of Nup358 (see below), suggesting that not all importin 7-cargos require this nucleoporin for efficient import.

In a previous study, we showed that different regions in Nup358 are involved in nuclear import of different substrates whose transport is strongly promoted by this nucleoporin [24]. We now used the same approach and expressed siRNA-resistant N-terminal fragments of Nup358 containing the NPC-anchor region together with hTERT-GFP in cells where endogenous Nup358 had been depleted. The Nup358-fragments could be visualized with an antibody against their HA-tag. Endogenous Nup358, on the other hand, was detected using an antibody directed against the C-terminal region of the nucleoporin, which is not present in the HA-tagged fragments. Figs. 4B, C show that the HA-Nup358-fragment 1–1306 is not able to rescue nuclear import of hTERT-GFP in Nup358-depleted cells. In our previous study this fragment clearly did support nuclear import of the Nup358-dependent cargo protein DBC-1 [24]. Two longer fragments of Nup358 (HA-Nup358 1–1810 and 1–2148), by contrast, allowed efficient import of hTERT-GFP into the nucleus in Nup358-depleted cells. These results demonstrate that the inhibition of nuclear import of hTERT-GFP in siRNA-treated cells does not result from off-target effects, as transport can be rescued by the expression of Nup358-fragments from siRNA-insensitive plasmids. They also suggest that the zinc finger region in Nup358, which is present in the 1-1810- but not the 1-1306-fragment, plays an important role in nuclear import of hTERT. We therefore tried to co-immunoprecipitate either endogenous Nup358 or Nup358-fragments with GFP-hTERT, using the GFP-trap (compare Fig. 3B). Using this approach, however, we were unable to detect specific binding of the nucleoporin to hTERT, suggesting that the putative interaction is rather transient and weak and/or mediated by an unknown adaptor (data not shown).

### Importin 7 and Nup358 Cooperate in Nuclear Import of hTERT-GFP

**Figure 5:**
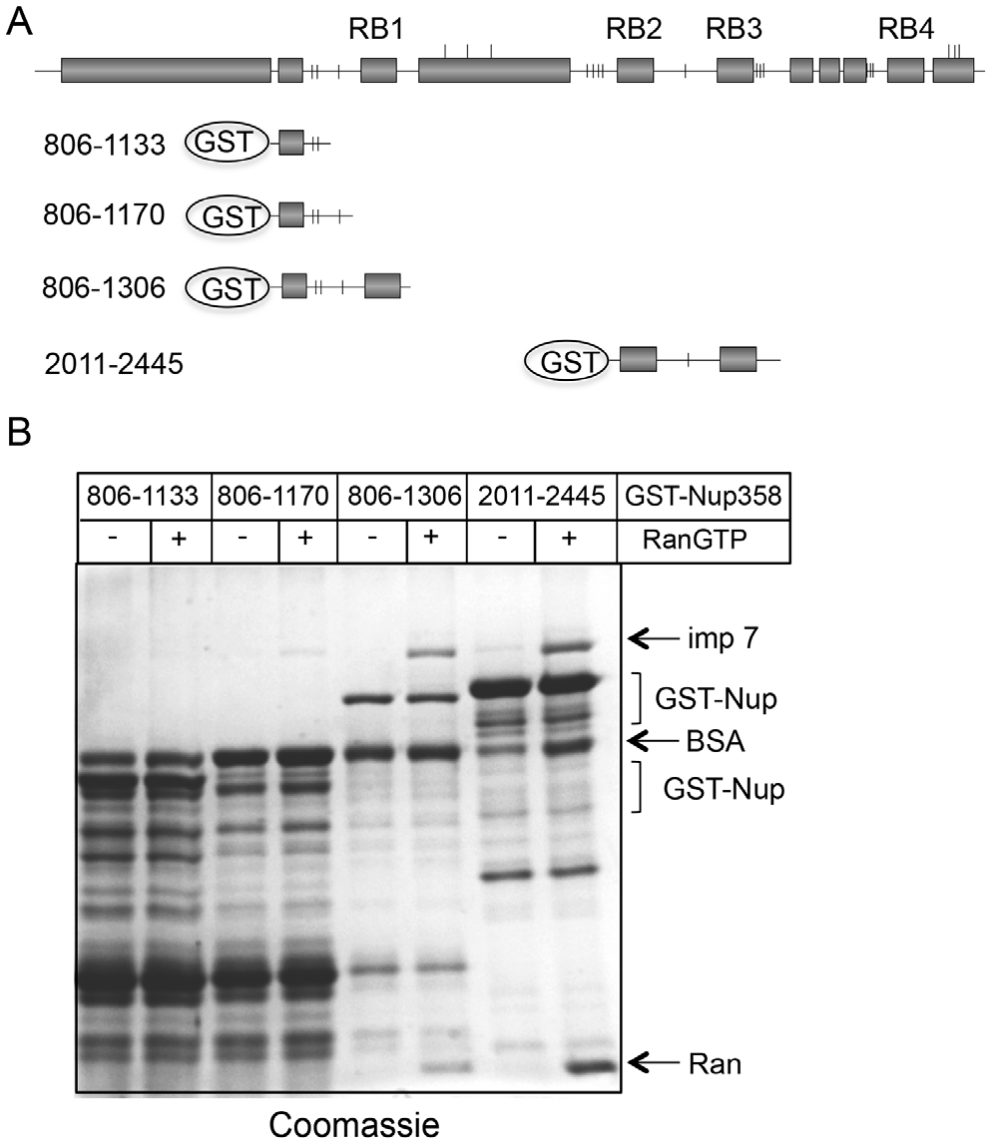
RanGTP-dependent binding of importin 7 to Nup358. A, Schematic representation of GST-Nup358 fragments (compare Fig. 4A). B, Pull-down experiment. Nup358-fragments were immobilized on beads and incubated with importin 7 in the absence or presence of Ran that had been loaded with GTP. Interacting proteins were analyzed by SDS-PAGE, followed by Coomassie-staining. Fragments 806–1133 and 806–1170 were prone to degradation. The hydrophobic FG-motifs can affect the mobility of protein fragments.

We next addressed the question of how Nup358 and importin 7 operate together to promote nuclear import of hTERT-GFP. The nucleoporin could have a direct function, binding the import substrate or the receptor-substrate complex prior to translocation across the nuclear pore. Alternatively (or in addition), Nup358 could function during the recycling of importin 7 from the nucleus back to the cytoplasm, as suggested for importin β [30], which is targeted to Nup358 by RanGTP [38]. In immunoprecipitation experiments using an antibody against Nup358, we were able to co-precipitate importin β but not importin 7 (data not shown). This negative result may reflect a relatively high concentration of importin β in HeLa cells, compared to importin 7. We therefore added recombinant importin 7 to the immunoprecipitate, in the absence or presence of RanQ69L-GTP. Binding of importin 7 to Nup358 was only detected in the presence of RanGTP, suggesting that the Ran-binding domains of the nucleoporin are involved in the interaction (data not shown). In a similar approach, we used recombinant proteins and immobilized GST-tagged fragments of Nup358 on beads and analyzed binding of His-tagged importin 7 in the absence or presence of RanGTP. As shown in Fig. 5, two Nup-fragments containing Ran-binding sites (GST-Nup358 806-1306 and 2011-2445) interacted with importin 7 in a RanGTP-dependent manner. Two other Nup358-fragments lacking Ran-binding sites (GST-Nup358 806–1133 and 806–1170), by contrast, showed no or only background binding of the import receptor. These results suggested that importin 7 can interact with Nup358 during recycling from the nucleus, i.e. via RanGTP bound to the import receptor. Unfortunately, we were unable to test direct interactions of hTERT or hTERT-importin 7 complexes with Nup358, resulting from the difficulties to express hTERT in bacteria.

**Figure 6:**
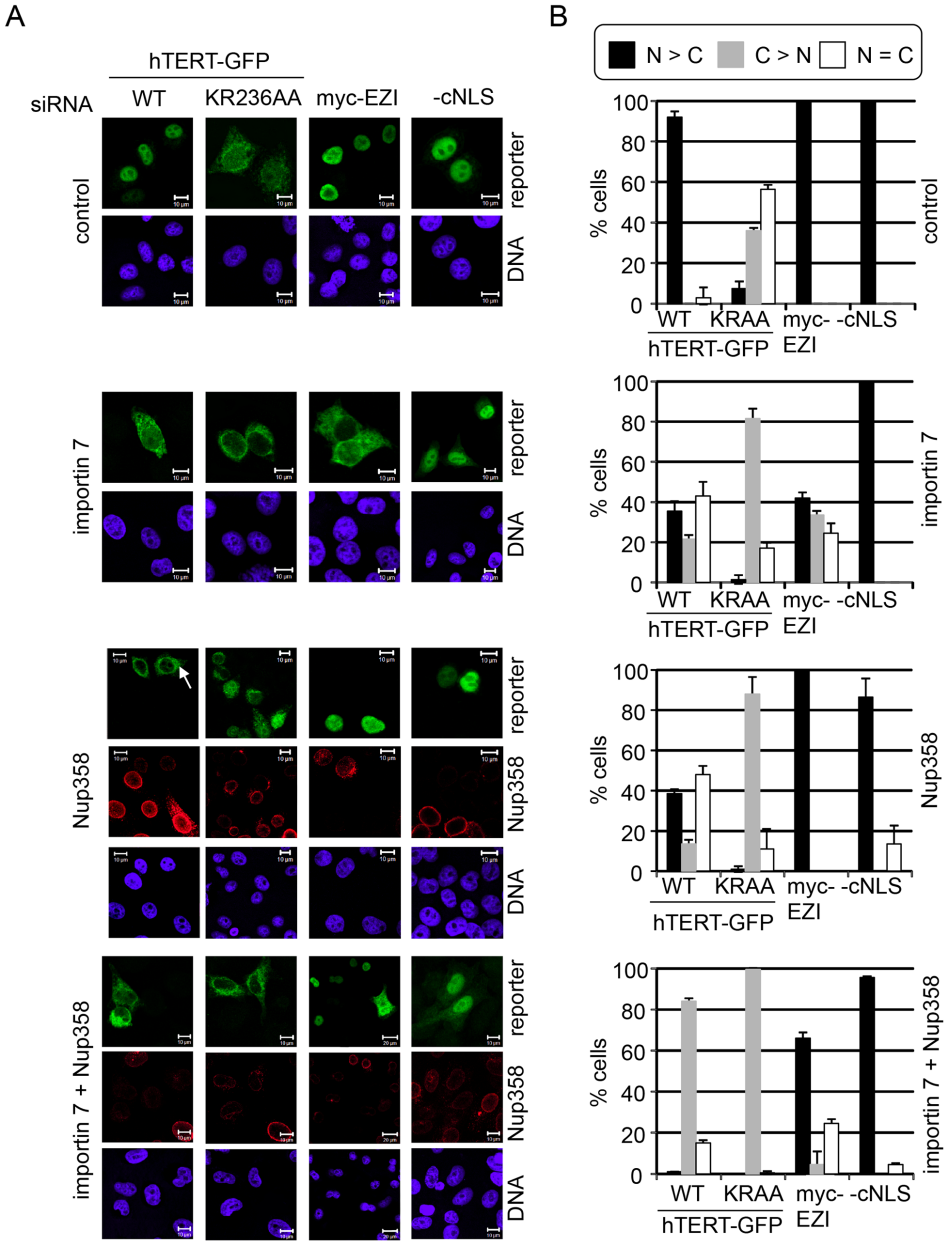
Nup358 and importin 7 cooperate in nuclear import of hTERT-GFP. A, HeLa cells were treated with control siRNA or siRNAs against importin 7, Nup358 or both, and transfected with plasmids coding for wild-type hTERT-GFP (WT), the NLS-mutant (KR236AA = K236AR237A), myc-EZI or NES-GFP_2_-cNLS (-cNLS), as indicated. Cells were fixed, subjected to indirect immunofluorescence to detect myc-EZI or Nup358 (where appropriate) and analyzed by fluorescence microscopy. Bars, 10 µm. White arrow, cell with reduced level of endogenous Nup358 and with hTERT-GFP in the cytoplasm. B, Quantification of the results in A. Error bars depict the standard deviation of the mean from at least three independent experiments.

Finally, we analyzed the effects of co-depletion of importin 7 and Nup358 on nuclear import of wild-type hTERT-GFP and of the import-restricted mutant KR236AA. As shown in Fig. 6, this mutant form of hTERT was largely excluded from the nucleus in either importin 7-, Nup358- or double-depleted cells, whereas mock-treated control cells allowed reduced but significant levels of nuclear import (compare Fig. 1). For wild-type hTERT-GFP, on the other hand, co-depletion of importin 7 and Nup358 had an additive or even synergistic effect on nuclear import, as the reporter was largely detected in the cytoplasm in more than 80% of the cells. For EZI, our importin 7-dependent control cargo, the double-depletion did not further reduce nuclear accumulation as compared to importin 7-depleted cells. In contrast, depletion of importin 7 alone resulted in stronger inhibition of nuclear accumulation of EZI, probably due to the lower concentrations of the import receptor in the singly depleted cells. Accordingly, immunoblot analysis showed that the residual levels of targeted proteins were lower when only single siRNAs were used in the experiment (data not shown).

We also tried to rescue import defects in Nup358-depleted cells by overexpression of HA-tagged importin 7. In contrast to certain cargos whose cognate import receptors become rate-limiting for nuclear import in the absence of the nucleoporin [29], [30], transport of hTERT-GFP into the nucleus was not enhanced by overexpression of importin 7 in cells lacking Nup358 (data not shown). Together, these results suggest a tight cooperation of the soluble import receptor importin 7 and the stationary NPC-component Nup358 in nuclear import of hTERT.

## Discussion

Extra-nuclear functions of telomerase have been reported [39]–[41], but a proper nuclear localization of the enzyme is a prerequisite for its best-understood activity in maintaining the ends of linear chromosomes. In a recent study, Chung et al. [21] described an NLS in the N-terminal region of hTERT (amino acids 222–240). hTERT-fragments lacking this N-terminal region were found in the cytoplasm, and the authors concluded that the N-terminal NLS in hTERT is the only sequence that mediates nuclear import. They also demonstrate that the NLS is important for hTERT to immortalize human foreskin fibroblast cells. In our experimental system, C-terminal and N-terminal fragments lacking the identified NLS accumulated in the nucleus as well (Fig. 1B), suggesting that additional pathways for nuclear import might exist. One possibility is that in our HeLa cells, hTERT-fragments interact with either full-length endogenous hTERT or with certain hTERT-binding proteins that promote nuclear import by a piggyback mechanism (for review see [2]). Alternatively, the fragments could enter the nucleus by passive diffusion and be retained there upon binding to chromatin. We therefore decided to focus on full-length proteins for our further analysis of hTERT nuclear import. In our study, we confirmed the importance of the basic amino acid at position 236–237 of the human protein for efficient nuclear import. Overall, TERT sequences from different species are not very well conserved (e.g. 62.4% sequence identity between mouse and man). The region surrounding the identified NLS in particular is rather diverse compared to, for example, the very N-terminal part of the protein (Fig. S2). The characteristic basic residues at amino acid position 236–237, however, are conserved between mammals, suggesting that this is a functionally important motif. Furthermore, we identified a related sequence at aa 649–650 that contributes to nuclear accumulation of hTERT (Fig. 1 C–E). These sequences with characteristic KR-motifs therefore seem to mediate transport of full-length endogenous protein into the nucleus. In addition, nuclear import of certain splice variants of hTERT that lack telomerase activity [42] contain these motifs and their nuclear import could therefore depend on these NLSs. It remains to be investigated, how the individual NLSs affect the biological activity of telomerase, i.e. telomere shortening and immortalization. Of note, hTERT S227A/7A (containing a mutated NLS) sustained cell growth over a period >150 days, similar to the wild type protein [21]. Similarly, cell lines expressing hTERT with mutations in the same region (N(236)AAIRS) were able to bypass crisis and were considered immortal [43]. These results indicate that the newly identified NLS (aa 649–650) might play an important role in cell immortalization.

Being characterized as a bipartite NLS, the identified sequence (aa 222–240) implied the classical importin α/β -dimer as an import receptor for hTERT [21]. Using siRNA-mediated depletion of several nuclear import receptors, however, we showed that the transport receptor importin α/β does not play a major role in hTERT-import, as depletion of importin β inhibited import of a cNLS-containing reporter protein but not that of full-length hTERT. Instead, we found that importin 7, also known as Ran-binding protein 7 (RanBP7, [44]), a member of the importin β-superfamily that is involved in nuclear import of histones, ribosomal proteins and a few other proteins [15], [17], [45], [46], plays a crucial role in transport of hTERT into the nucleus. Importantly, nuclear import of hTERT and of the established importin 7-substrate EZI could be rescued by the expression of siRNA-insensitive, HA-tagged importin 7, demonstrating the specificity of our approach. Importin 7 could either function on its own or in combination with importin β, as dimers of the two receptor proteins are involved in nuclear import of certain substrates [47]. Since the depletion of importin β had essentially no effect on the nuclear accumulation of hTERT-GFP, importin 7 is likely to function on its own in nuclear import of telomerase. To demonstrate direct binding between cargos and import receptors, purified proteins are required. Unfortunately, all our efforts to express full-length hTERT in bacteria or in insect cells remained unsuccessful.

In the majority of nuclear import pathways, RanGTP plays a crucial role as it binds to all members of the importin β-family [11], leading to dissociation of import complexes in the nucleus. Previous findings suggested a low affinity of importin 7 for RanGTP, compared to other import receptors [47]–[49]. Our observation that RanQ69L-GTP did not significantly reduce the co-immunoprecipitation of hTERT-GFP and importin 7 (Fig. 3B) fits very well to these findings. Perhaps, importin 7-substrates require additional nuclear protein factors and/or binding to DNA for dissociation from their import receptor. Interestingly, the DNA-binding domain of TERT is in the N-terminal part of the protein, i.e. in close proximity to the major NLS [50]. Other possible dissociation factors are nucleolin, an abundant nuclear phosphoprotein that binds hTERT and affects its nuclear localization [51] and the RNA-component TERC. Again, purified proteins will be required for a more detailed characterization of factors that are involved in the dissociation of hTERT-containing import complexes.

**Figure 7:**
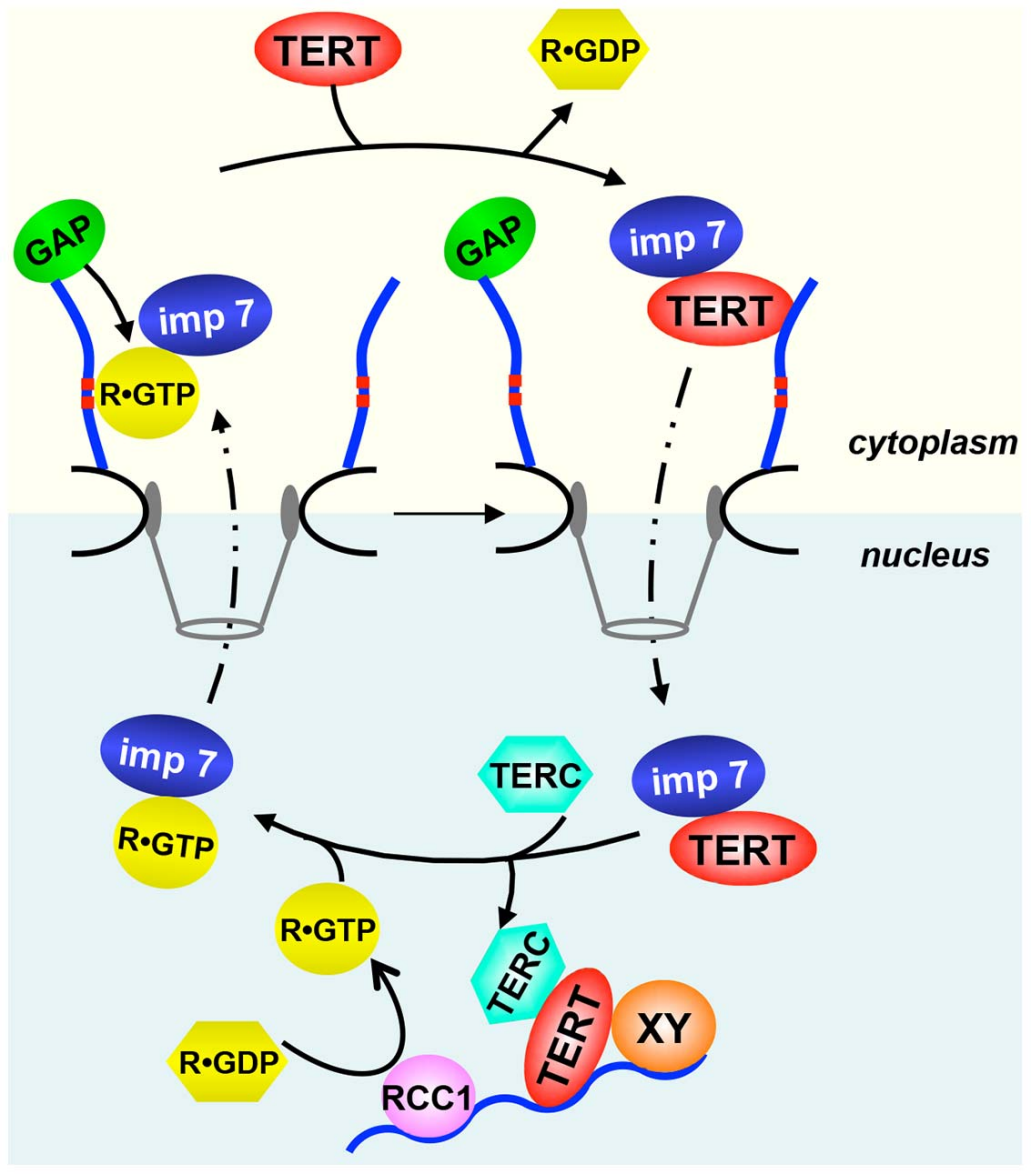
Model for importin 7- and Nup358-assisted import of hTERT. Cytoplasmic hTERT (TERT) interacts with Nup358, the major component of the cytoplasmic filaments of the NPC (blue rods). Upon binding of importin 7, hTERT is translocated across the NPC. In the nucleus, several mechanisms could promote the dissociation of the import complex: binding of RanGTP (R•GTP), which is generated in the vicinity of chromatin-bound RCC1, to importin 7; binding of hTERT to DNA; interaction of TERC, the RNA-component of telomerase with hTERT; accessory factors (XY) that bind to hTERT. The resulting importin 7/RanGTP complex then recycles back to the cytoplasmic side of the NPC, where it is captured by the Ran-binding sites of Nup358 (two of them are shown as red boxes). Finally, Nup358-associated RanGAP promotes GTP-hydrolysis on Ran, resulting in free RanGDP (R•GDP) and importin 7, which is kept in the vicinity of the NPC to initiate a new round of import.

We have previously described Nup358 as a nucleoporin that affects nuclear accumulation of a subset of cellular proteins [24]. The identified proteins used the major nuclear transport receptors importin α/β or transportin for import into the nucleus. In this study we now identified hTERT as an importin 7 cargo whose nuclear accumulation is strongly reduced in Nup358-depleted cells. As seen before for other substrates, the import defect does not simply result from the recruitment of a specific import receptor, as EZI, an established importin 7- cargo, was not affected by the depletion of the nucleoporin. Furthermore, a structure-function analysis showed that specific regions in Nup358 are involved in nuclear import of Nup358-dependent cargos. Whereas the first 1170 amino acids of the Nup358 sequence were sufficient to rescue nuclear import of the importin α/β -cargo DBC-1 in cells depleted of the endogenous protein [24], much longer fragments (aa 1–1810 or 1–2148; Fig. 4) were required to rescue transport of hTERT. Although we were unable to demonstrate a direct interaction of hTERT with Nup358 (as we did for other import cargos; [24]), it is possible that the protein binds to a specific region of the nucleoporin, e.g. the zinc finger domain, either directly or via an adaptor. Importin 7 is expected to bind RanGTP in the nucleus, translocate back to the cytoplasm through the nuclear pore and associate with the Ran-binding domain of Nup358. Indeed, our results demonstrate that such a RanGTP-dependent interaction of importin 7 with Nup358 is possible (Fig. 5). After GTP-hydrolysis on Ran, which is promoted by Nup358-associated RanGAP [19], importin 7 would be free for a new round of import and could immediately bind to an appropriate cargo in its direct vicinity. By such a mechanism that is linked to the recycling of the import receptor, its active concentration at the NPC is increased, as we showed previously for importin β [30]. This effect alone, however, cannot explain reduced nuclear import of hTERT in cells depleted of Nup358, since the phenotype could not be rescued by overexpression of importin 7. Hence, efficient nuclear import of hTERT seems to require another mechanism and could be facilitated by its interaction with Nup358, as suggested before for other cargos [24]. A model of nuclear import of hTERT is shown in Fig. 7.

Together, our study provides important information about nuclear import of hTERT at the level of the cargo, the soluble import receptor and the nuclear pore complex. It will be interesting to analyze whether importin 7 and Nup358 play a role in maintaining high levels of nuclear TERT-activities and, thus, to prevent chromosomal damage. Interestingly, elevated levels of importin 7 transcripts were found in various tumor cells [52], [53], suggesting that the nuclear transport pathway of hTERT might be a potential target for future drug development.

## Supporting Information

Figure S1 Importin 7 functions as an import receptor for hTERT. HeLa cells expressing hTERT-GFP that had been treated with either control siRNAs or with siRNAs against importin 7 were analyzed for the dynamics of nuclear import of the reporter protein by FLIP. The graphs show the mean loss in fluorescence in three independent experiments, analyzing a total of 45 cells per condition. This is the same experiment as the one shown in Fig. 3A, including error bars.
(TIF)

Figure S2 Alignment of the N-terminal region of five mammalian TERT-sequences (amino acids 1-349 for the human sequence). The NLS-region of the human sequence and the conserved basic residues are depicted in red. Asterisks, identical residues; colons, conservative changes.
(TIF)
