# Supplementary figures and images for: Importin 7 and Nup358 Promote Nuclear Import of the Protein Component of Human Telomerase
Source: PLoS One. 2014 Feb 20;9(2):e88887. doi: 10.1371/journal.pone.0088887 (PMC3930611; doi:10.1371/journal.pone.0088887)

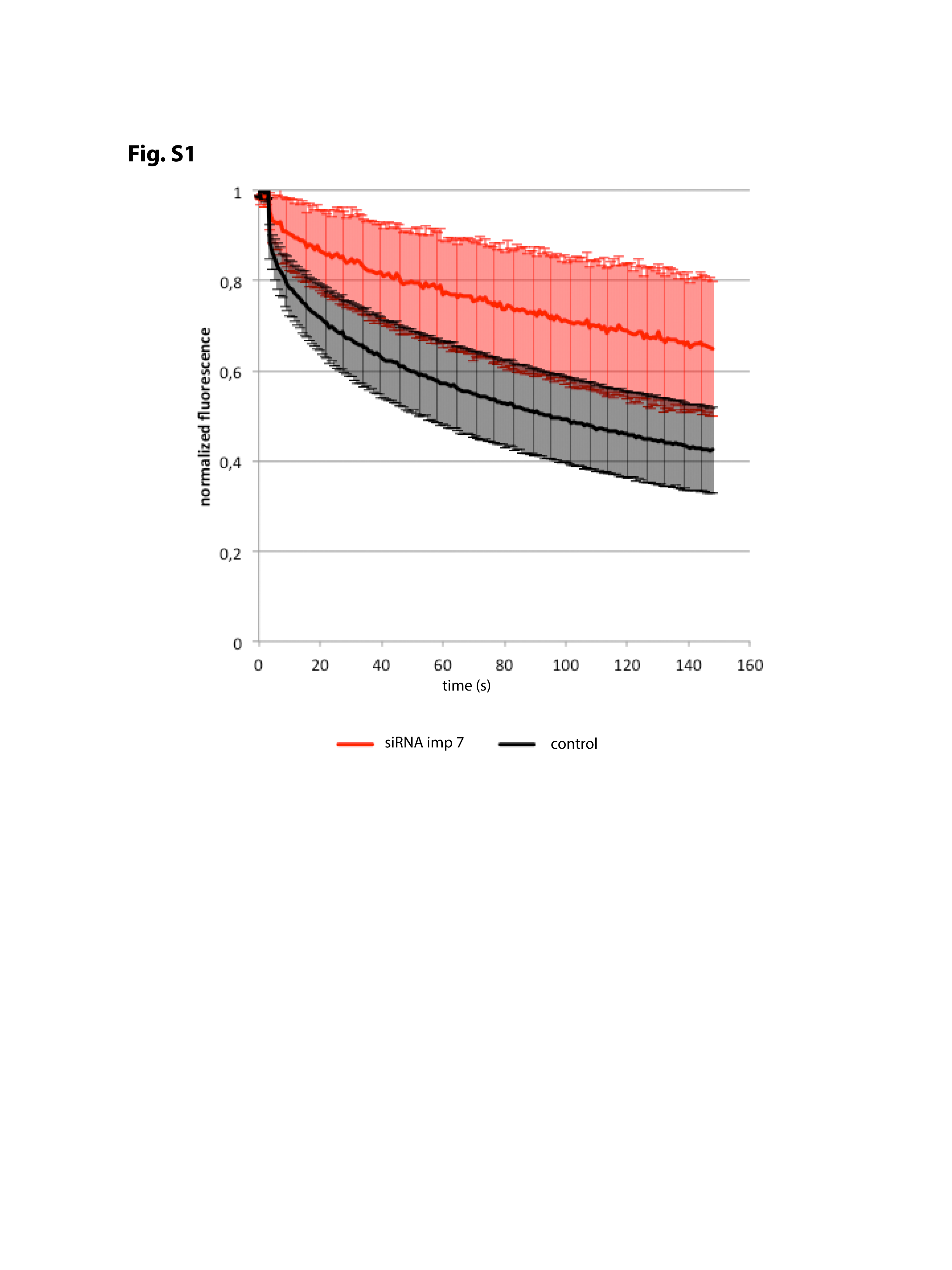

Supplement: Figure S1 — Importin 7 functions as an import receptor for hTERT. HeLa cells expressing hTERT-GFP that had been treated with either control siRNAs or with siRNAs against importin 7 were analyzed for the dynamics of nuclear import of the reporter protein by FLIP. The graphs show the mean loss in fluorescence in three independent experiments, analyzing a total of 45 cells per condition. This is the same experiment as the one shown in Fig. 3A, including error bars. (TIF) [file pone.0088887.s001.tif]

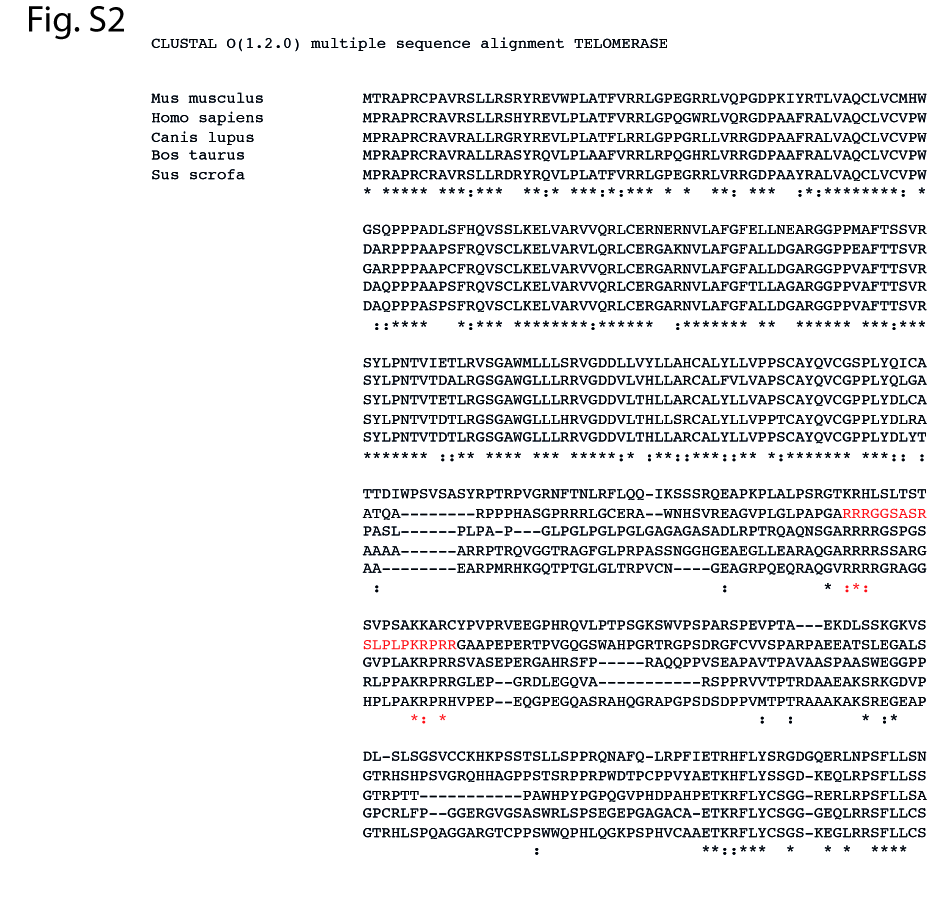

Supplement: Figure S2 — Alignment of the N-terminal region of five mammalian TERT-sequences (amino acids 1-349 for the human sequence). The NLS-region of the human sequence and the conserved basic residues are depicted in red. Asterisks, identical residues; colons, conservative changes. (TIF) [file pone.0088887.s002.tif]
